# The prognostic and immunological effects of ZBTB7C across cancers: friend or foe?

**DOI:** 10.18632/aging.202955

**Published:** 2021-05-04

**Authors:** Xuenuo Chen, Zhongxiang Jiang, Zhijian Wang, Zheng Jiang

**Affiliations:** 1 Department of Gastroenterology, The First Affiliated Hospital of Chongqing Medical University, Chongqing, China

**Keywords:** ZBTB7C, pancancer, survival, biomarker, Immune infiltration

## Abstract

As an important transcription factor, zinc-finger and BTB domain-containing 7B (ZBTB7C) plays an important role in a variety of tumors. However, its relationship with human immunity is unclear. This article aims to study its differential expression and survival across cancers and explore the relationships between its differential expression and the tumor microenvironment and immune cell infiltration. In this study, we used R software to process The Cancer Genome Atlas (TCGA) data and explored the expression pattern and prognostic value of ZBTB7C across cancers. Next, we comprehensively explained the important role of ZBTB7C in several tumor types in terms of tumor mutational burden (TMB), microsatellite instability (MSI) and immune cell infiltration. In general, the expression level of ZBTB7C in tumor tissues was lower than that in normal tissues. Highly expressed ZBTB7C was beneficial to the survival of patients with colon adenocarcinoma (COAD), lymphoid neoplasm diffuses large B cell lymphoma (DLBC), esophageal carcinoma (ESCA) and mesothelioma (MESO). Multivariate analysis showed that the expression of ZBTB7C was an independent prognostic factor in COAD and MESO. In COAD, the expression of ZBTB7C was positively correlated with both TMB and MSI. In colorectal cancer (CRC), there was a significant positive correlation between ZBTB7C expression and immune cell infiltration, especially the infiltration of mast cells and B cells. In conclusion, ZBTB7C can be used as a potential therapeutic target across cancers and is related to immune cell infiltration.

## INTRODUCTION

With the rapid growth and aging of the world’s population, cancer has become increasingly prominent. Cancer will become the main cause of death in the world within the 21st century and the single most important obstacle to increasing life expectancy [1]. The occurrence and development of tumors are complex processes with multiple stages, multiple genes and multiple components [2]. Increasing evidence shows that microsatellite instability (MSI), tumor mutational burden (TMB), and the tumor microenvironment (TME) (especially the tumor immune microenvironment) play important roles in the occurrence and development of tumors [3, 4]. Immunotherapy and targeted therapy are the fourth most common therapies for cancer therapy after surgery and radiotherapy. They play an important role in improving the therapeutic effect of malignant tumors, prolonging the survival time and reducing the side effects of radiotherapy and chemotherapy. As a new therapeutic mode, tumor immunity and its targeted therapy have received great attention.

POK/ZBTB family proteins are part of a key transcription factor family and involved in a variety of biological processes, including but not limited to regulation of immunity and tumor development [5–8]. Zinc-finger and BTB domain-containing 7B (ZBTB7C), a member of the ZBTB transcription factor family, has reduced or absent expression in most cervical cancer cell lines and has been proven to inhibit the proliferation of cervical cancer cells [9]. The ZBTB7C gene has recently been confirmed to be an independent prognostic factor for colorectal cancer (CRC) and a tumor suppressor gene involved in the occurrence and development of colorectal cancer [10]. However, ZBTB7C in clear cell renal cancer cells can function as a proto-oncogene to stimulate the rapid proliferation of tumor cells [11]. Although the important role of ZBTB7C in tumors has been hinted at, we have not yet systematically and comprehensively determined it. The expression patterns and mechanisms of action of ZBTB7C in different tumor cells are significantly different. Therefore, it is not clear whether ZBTB7C is a friend or an enemy across cancers.

In this study, first, based on transcriptome, mutation and clinical data from The Cancer Genome Atlas (TCGA) database, we carried out pancancer analysis of ZBTB7C, including analysis of its differential expression between cancer and adjacent tissues, survival analysis, and correlation analysis of TMB and MSI. Then, we studied the relationship between the infiltration of 24 kinds of immune cells and ZBTB7C in CRC and used the single-sample gene set enrichment analysis (ssGSEA) algorithm to determine the immune profile. Finally, RT-qPCR and immunohistochemistry (IHC) were used to verify our conclusion. The findings suggest that ZBTB7C may be a new potential therapeutic target and is related to immune cell infiltration.

## RESULTS

### ZBTB7C expression across cancers

**Figure 1:**
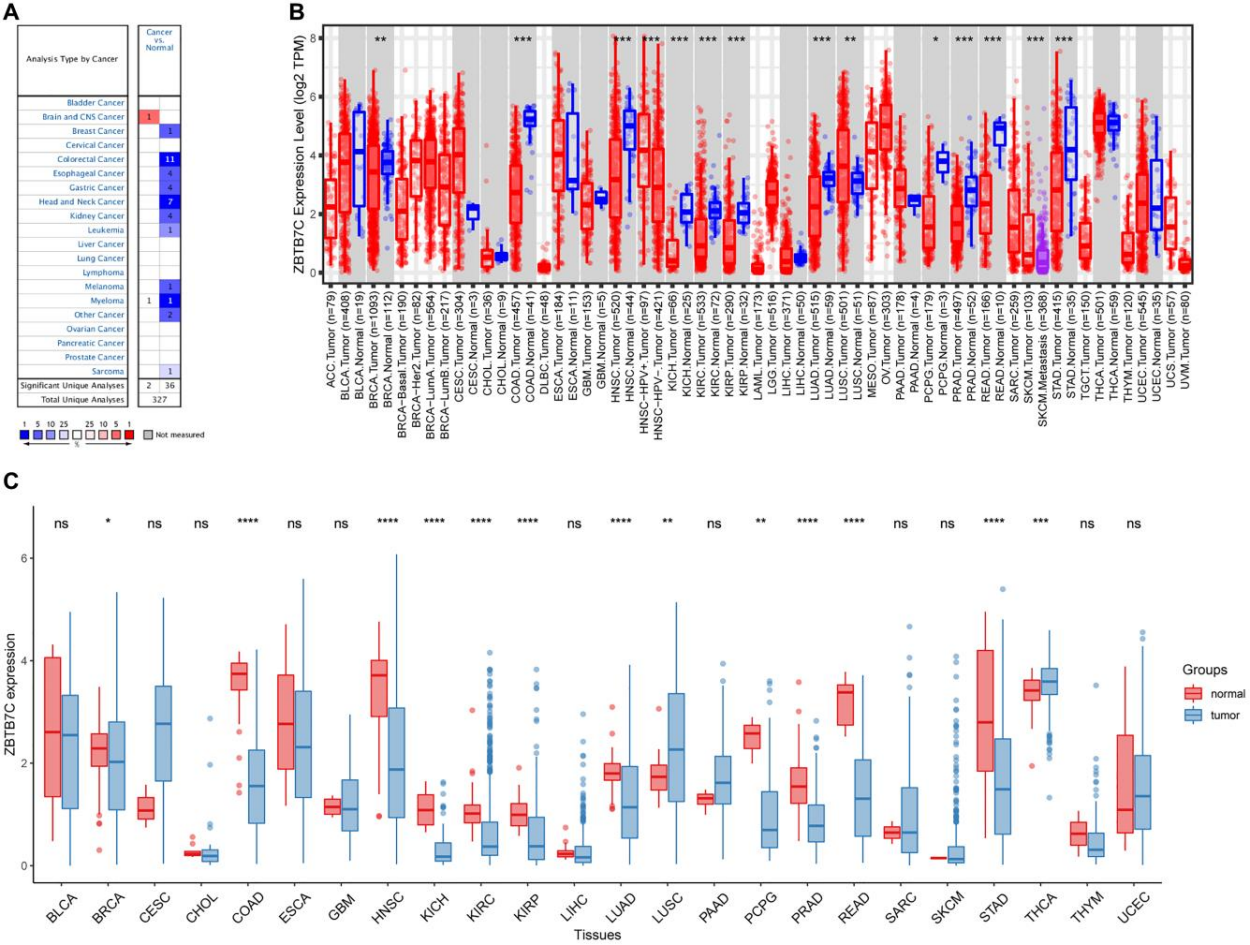
**The expression level of ZBTB7C in human cancers.** (**A**) Oncomine database. (**B**) TIMER database. (**C**) TCGA database (^*^P < 0.05, ^**^P < 0.01, ^***^P < 0.001).

The different expression patterns of ZBTB7C in tumors and adjacent normal tissues are shown in Figure 1C. Compared with that in normal samples from TCGA, the expression of ZBTB7C mRNA in lung squamous cell carcinoma (LUSC), breast invasive carcinoma (BRCA), colon adenocarcinoma (COAD), head and neck squamous cell carcinoma (HNSC), kidney chromophobe (KICH), kidney renal clear cell carcinoma (KIRC), and kidney renal papillary cell carcinoma (KIRP) was increased. In contrast, ZBTB7C mRNA expression was significantly reduced in lung adenocarcinoma (LUAD), pheochromocytoma and paraganglioma (PCPG), prostate adenocarcinoma (PRAD), rectal adenocarcinoma (READ), stomach adenocarcinoma (STAD) and thyroid carcinoma (THCA) samples compared with that in normal samples. To further evaluate the expression of ZBTB7C across cancers, we used the Oncomine database and the TIMER database to verify and supplement our results (Figure 1A, 1B). We obtained similar analysis results. Compared with adjacent tissues, most tumor tissues had significantly lower expression of ZBTB7C.

### The prognostic value of ZBTB7C in tumors

**Figure 2:**
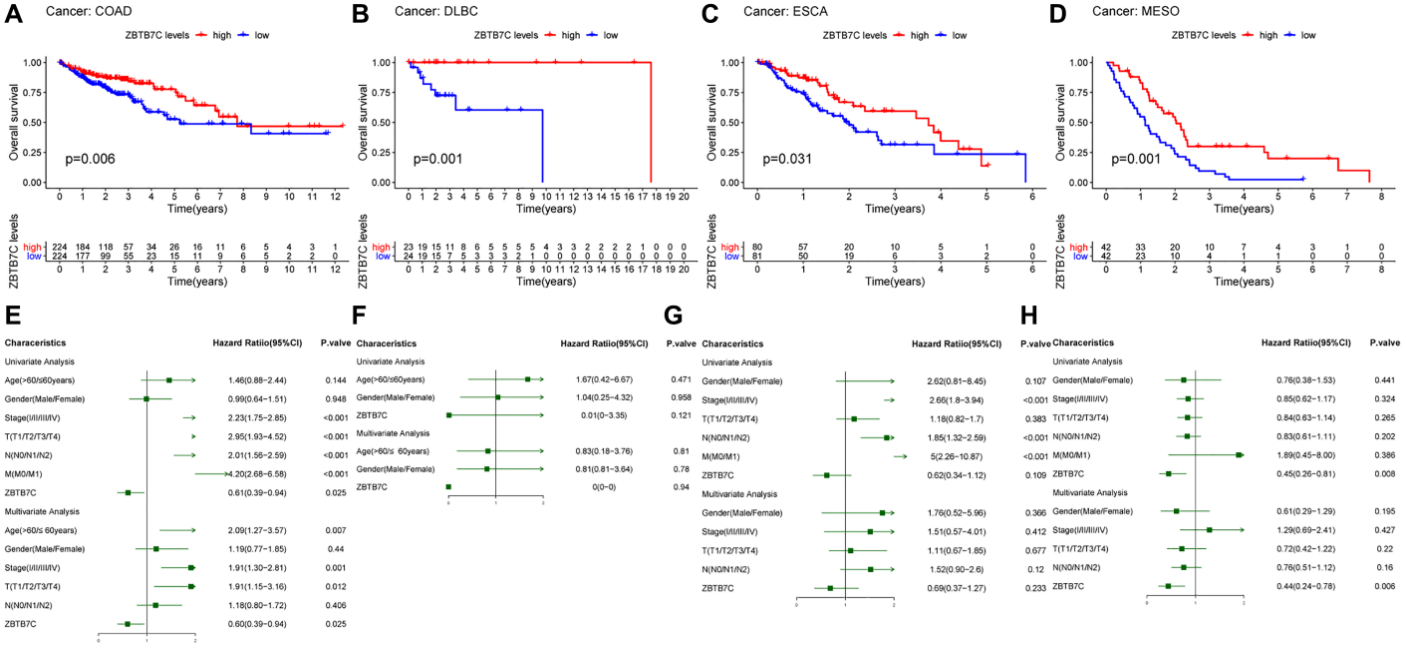
**Survival analysis of ZBTB7C in different tumors.** OS and Cox regression analyses of (**A**, **E**) colon adenocarcinoma (COAD), (**B**, **F**) lymphoid neoplasm diffuse large B-cell lymphoma (DLBC), (**C**, **G**) esophageal carcinoma (ESCA) and (**D**, **H**) mesothelioma (MESO). OS, overall survival.

Based on the TCGA raw data, Kaplan-Meier survival curves were constructed and used to evaluate the prognostic value of ZBTB7C across cancers. As shown in Figure 2A–2D, the expression of ZBTB7C was significantly correlated with the overall survival (OS) of 4 cancers: COAD (*P* = 0.006), DLBC (*P* = 0.001), ESCA (*P* = 0.031) and MESO (*P* = 0.001). Interestingly, low expression of ZBTB7C indicated a poor prognosis. Next, we conducted Cox proportional hazard regression analysis to study the potential of ZBTB7C expression as a prognostic biomarker for the above four tumors. Univariate analysis showed that the higher the expression of ZBTB7C was, the longer the OS of patients with COAD and MESO. In the multivariate analysis, ZBTB7C, as a tumor suppressor gene, was an independent prognostic factor in COAD (hazard ratio = 0.60; 95% confidence interval (CI), 0.39–0.94; *P* = 0.025; Figure 2E) and MESO (hazard ratio = 0.44); 95% CI, 0.24–0.78; *P* = 0.006; Figure 2H). Conversely, the multivariate analysis of DLBC (Figure 2F) and ESCA (Figure 2G) was negative.

### Correlation of ZBTB7C with TMB and MSI

**Figure 3:**
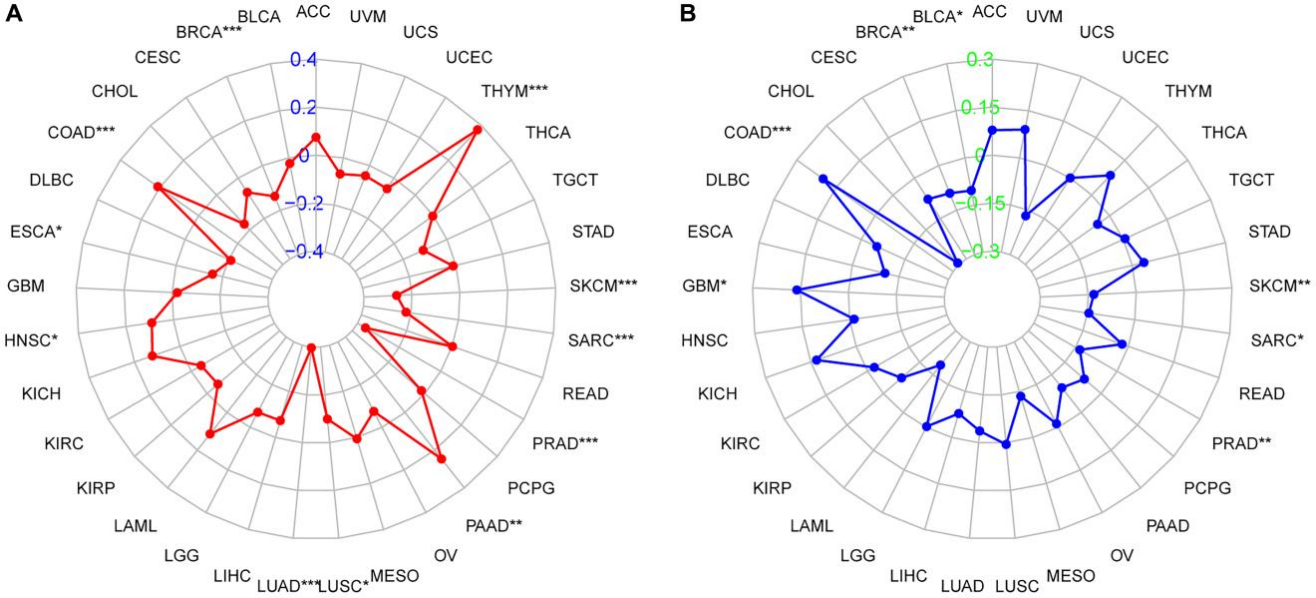
Radar chart of the correlation between ZBTB7C and (**A**) tumor mutational burden (TMB) and (**B**) microsatellite instability (MSI) in pancancer.

R software was used to calculate the correlation between ZBTB7C expression and TMB, and the Fmsb R package was used for visualization. The results showed that, on the one hand, the expression of ZBTB7C was negatively correlated with TMB in BRCA (*r* = -0.138, *p* < 0.001), ESCA (*r* = -0.157, *p* = 0.048), LUAD (*r* = -0.397, *p* < 0.001), LUSC (*r* = - 0.099, *p* = 0.029), PRAD (*r* = -0.362, *p* < 0.001), sarcoma (SARC) (*r* = -0.221, *p* < 0.001) and skin cutaneous melanoma (SKCM) (*r* = -0.264, *p* < 0.001). On the other hand, in COAD (*r* = 0.209, *p* < 0.001), HNSC (*r* = 0.091, *p* = 0.044), PAAD (*r* = 0.246, *p* = 0.002) and THYM (*r* = 0.376, *p* < 0.001), ZBTB7C was positively correlated with TMB (Figure 3A and Supplementary Table 1). Similarly, correlation analysis between ZBTB7C expression and MSI was performed according to the above method. In BLCA (*r* = -0.104, *p* = 0.036), BRCA (*r* = -0.093, *p* = 0.003), PRAD (*r* = -0.134, *p* = 0.003), SARC (*r* = -0.145, *p* = 0.021) and SKCM (*r* = -0.132, *p* = 0.004), ZBTB7C expression was negatively correlated with MSI; in COAD (*r* = 0.200, *p* < 0.001) and GBM (*r* = 0.161, *p* = 0.048), ZBTB7C expression was positively correlated with MSI (Figure 3B and Supplementary Table 2).

### Correlation between ZBTB7C expression and the CRC immune profile

**Figure 4:**
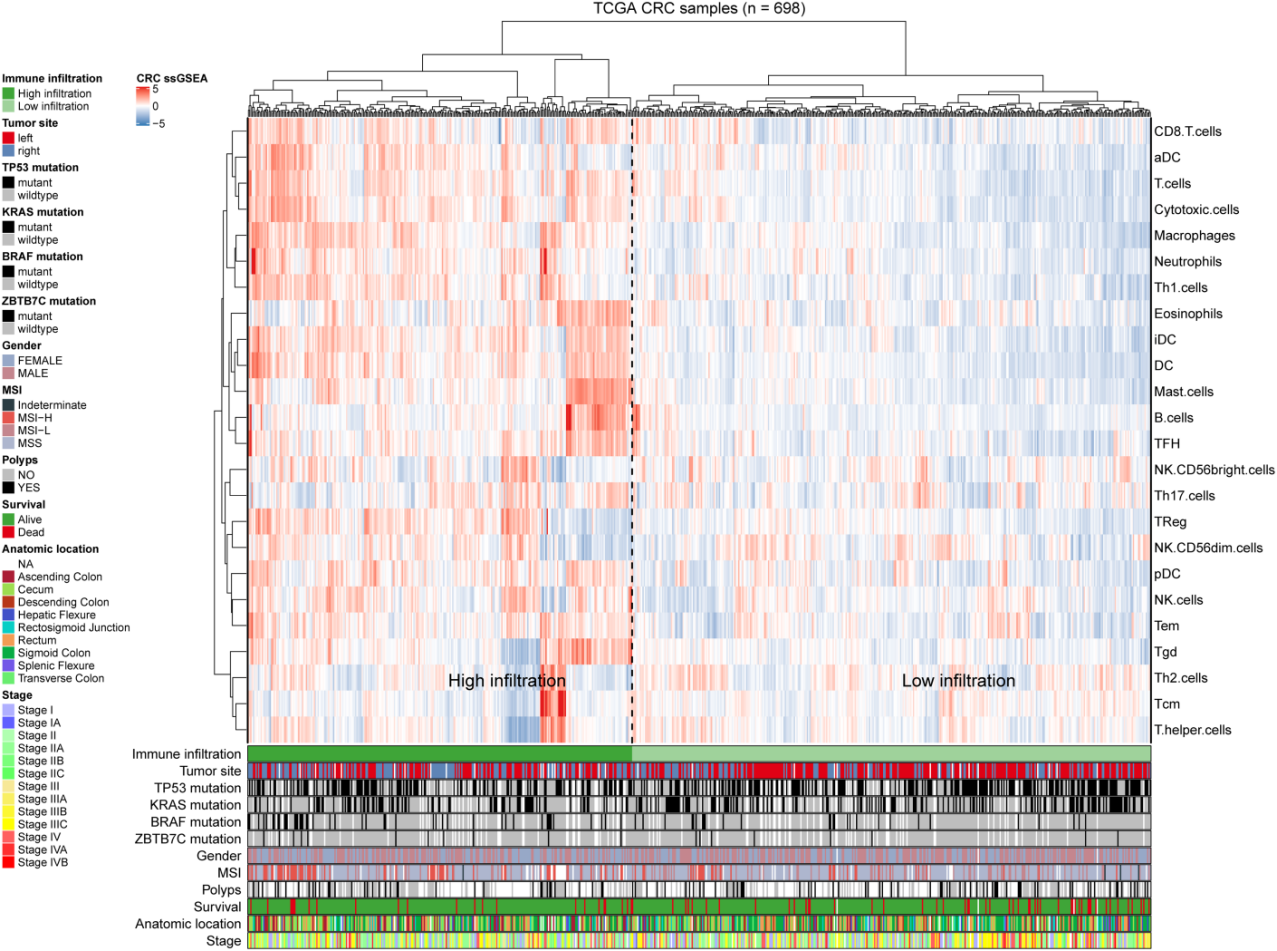
**Immune panorama of colorectal cancer.** Using single-sample gene set enrichment analysis scores from 24 immune cell types, 698 patients from The Cancer Genome Atlas cohort were clustered. The following icon shows the tumor site, the mutation statuses of BRAF, TP53, KRAS and ZBTB7C, metastasis, sex, MSI, polyps, survival rate, anatomical location and stage. Two different immune infiltration clusters are defined: high infiltration and low infiltration.

**Figure 5:**
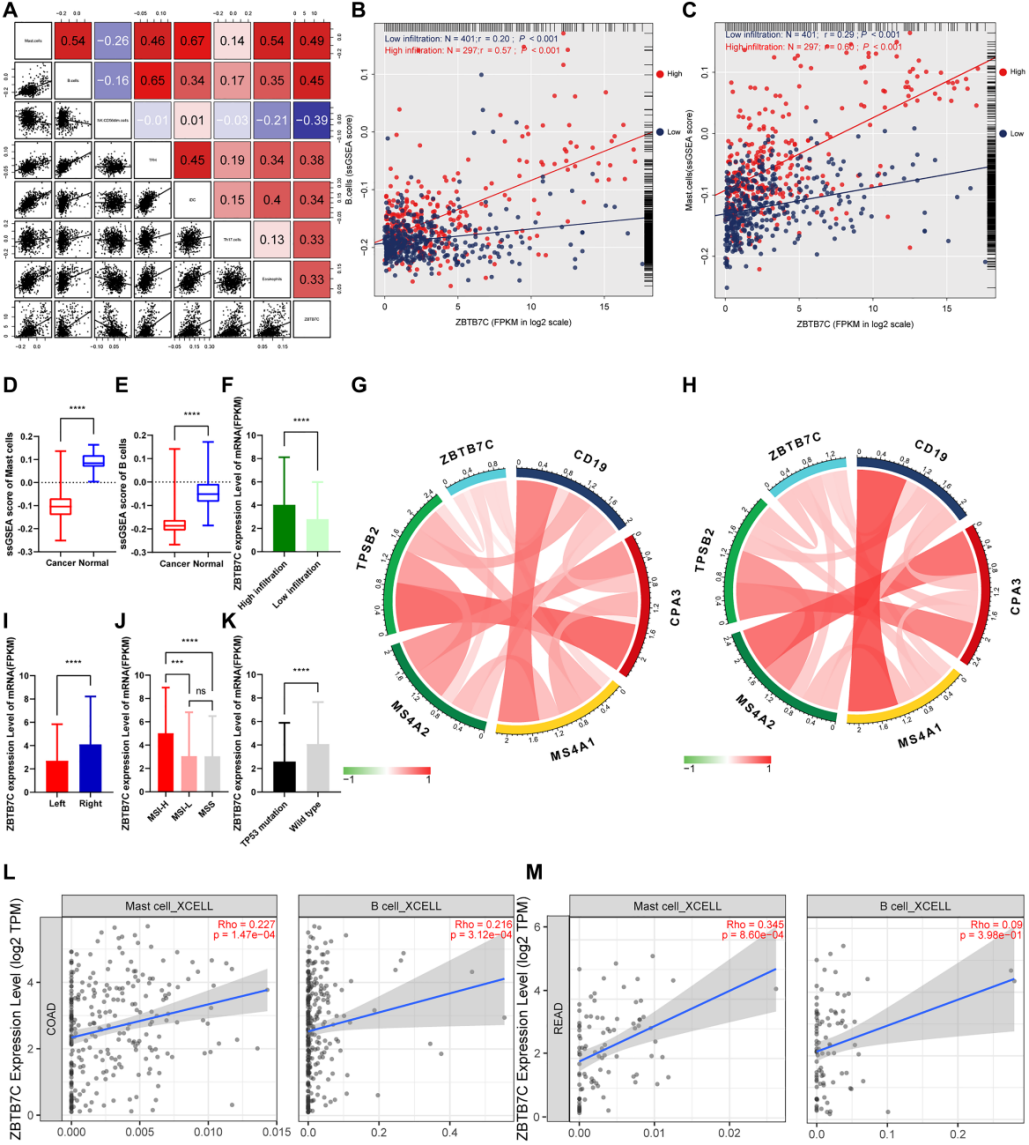
**ZBTB7C and immune infiltration in colorectal cancer.** (**A**) The top seven immune-infiltrating cells with the strongest correlation with ZBTB7C. (**B**–**C**) The correlation between (**B**) B cells and (**C**) mast cells and ZBTB7C using the ssGSEA method. (**D**–**E**) Differential expression of (**D**) mast cells and (**E**) B cells in colorectal cancer and adjacent tissues. (**F**–**K**) The differential expression of ZBTB7C in (**F**) the high and low infiltration groups, (**I**) left and right colorectal cancers, and (**K**) mutant and wild type TP53. (G, H) The correlation between ZBTB7C and immune markers in the (**G**) GEO and (**H**) TCGA databases. (**L**, **M**) Based on the TIMER database, the correlation between ZBTB7C and mast cells and B cells in (**L**) colon adenocarcinoma (COAD) and (**M**) rectal adenocarcinoma (READ).

To explore the potential relationship between ZBTB7C and infiltrating immune cells in CRC, we used ssGSEA [12] to determine the CRC immune profile (Figure 4). This analysis included 698 CRC patients. We calculated the correlation between ZBTB7C and 24 immune cells, stage, sex, tumor location, and mutation. According to the clustering analysis of the degree of immune cell infiltration, we divided the cohort into two subgroups: a group with high (*n* = 297) and a group with low (*n* = 401) infiltration. ZBTB7C had a higher expression level in the high infiltration group than in the low infiltration group (Figure 5F). The expression of ZBTB7C showed significant differences according to tumor location. ZBTB7C expression was higher in right-sided CRC than in left-sided CRC (Figure 5I). Consistent with the results of the previous MSI study, ZBTB7C expression was higher in the high MSI (MSI-H) group than in the microsatellite stable (MSS) group, and there was no difference between the low MSI (MSI-L) and MSS groups (Figure 5J). Finally, we found that the expression of ZBTB7C in the TP53 mutant group was decreased (Figure 5K). It is worth mentioning that ZBTB7C was positively correlated with 12 kinds of immune cells and negatively correlated with 2 kinds of immune cells (Table 1). Sorted by the absolute value of the correlation coefficient, the top seven immune cells were mast cells, B cells, natural killer (NK) CD56dim cells, T follicular helper (Tfh) cells, immature dendritic cells (iDCs), T helper 17 (Th17) cells and eosinophils (Figure 5A). We considered the top two cell types (mast cells and B cells) in further analyses. The correlation between B cell infiltration and ZBTB7C (*r* = 0.57, *p* < 0.001) was stronger in the high infiltration group than in the low infiltration group (*r* = 0.20, *p* < 0.001; Figure 5B). Mast cell infiltration showed a similar trend: the high infiltration group showed a stronger correlation between mast cell infiltration and ZBTB7C expression (*r* = 0.60, *p* < 0.001) than the low infiltration group (*r* = 0.29, *p* < 0.001; Figure 5C). At the same time, we found that mast cells (Figure 5D) and B cells (Figure 5E) showed low expression in colorectal cancer. The TIMER database verified this correlation in COAD (Figure 5L) and READ (Figure 5M).

**Table 1:** Correlations between ZBTB7C and immune cells in CRC.

**Cell type**	**Cor**	***P***
aDC	–0.047	0.211
B.cells	0.453	^***^
CD8.T.cells	0.209	^***^
Cytotoxic.cells	0.193	^***^
DC	0.226	^***^
Eosinophils	0.328	^***^
iDC	0.339	^***^
Macrophages	0.037	0.323
Mast.cells	0.491	^***^
Neutrophils	–0.048	0.203
NK.CD56bright.cells	0.166	^***^
NK.CD56dim.cells	–0.392	^***^
NK.cells	0	0.995
pDC	0.075	^*^
T.cells	0.227	^***^
T.helper.cells	–0.015	0.697
Tcm	–0.051	0.179
Tem	–0.017	0.649
TFH	0.381	^***^
Tgd	0.239	^***^
Th1.cells	–0.094	^*^
Th17.cells	0.334	^***^
Th2.cells	–0.026	0.489
TReg	–0.244	^***^

Abbreviations: CRC: colorectal cancer; aDC: activated dendritic cell; DC: dendritic cell; iDC: immature dendritic cell; pDC: plasmacytoid dendritic cell; Tcm: T central memory; Tem: T ffector memory; TFH: T follicular helper cell; Tgd: T gamma delta cell; TReg: regulatory T cells; Cor: R value of Spearman’s correlation. ^*^P < 0.05; ^**^P < 0.01; ^***^P < 0.001.

### The relationship between ZBTB7C expression and immune markers

**Figure 6:**
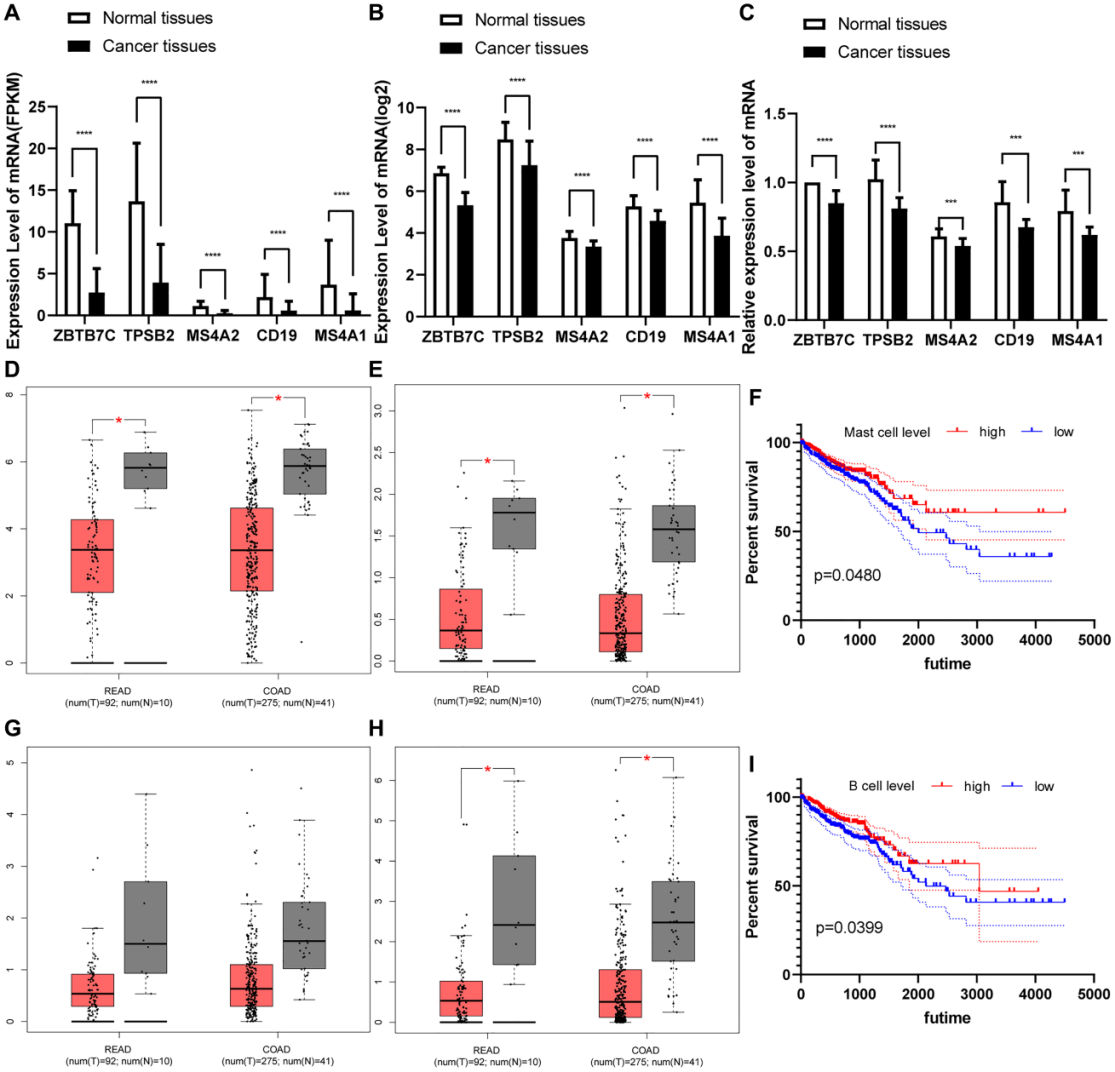
(**A**–**C**) The differential expression of ZBTB7C and immune markers in colorectal cancer and adjacent tissues. (**A**) From the TCGA. (**B**) From the GEO. (**C**) Seventeen pairs of colorectal cancer tissues and adjacent tissues from the First Affiliated Hospital of Chongqing Medical University. (**D**–**H**) Expression of (**D**) TPSB2, (**E**) MS4A2, (**G**) CD19 and (**H**) MS4A1 in cancer and adjacent tissues. (**F**, **I**) Survival curve of immune cells in colorectal cancer.

**Figure 7:**
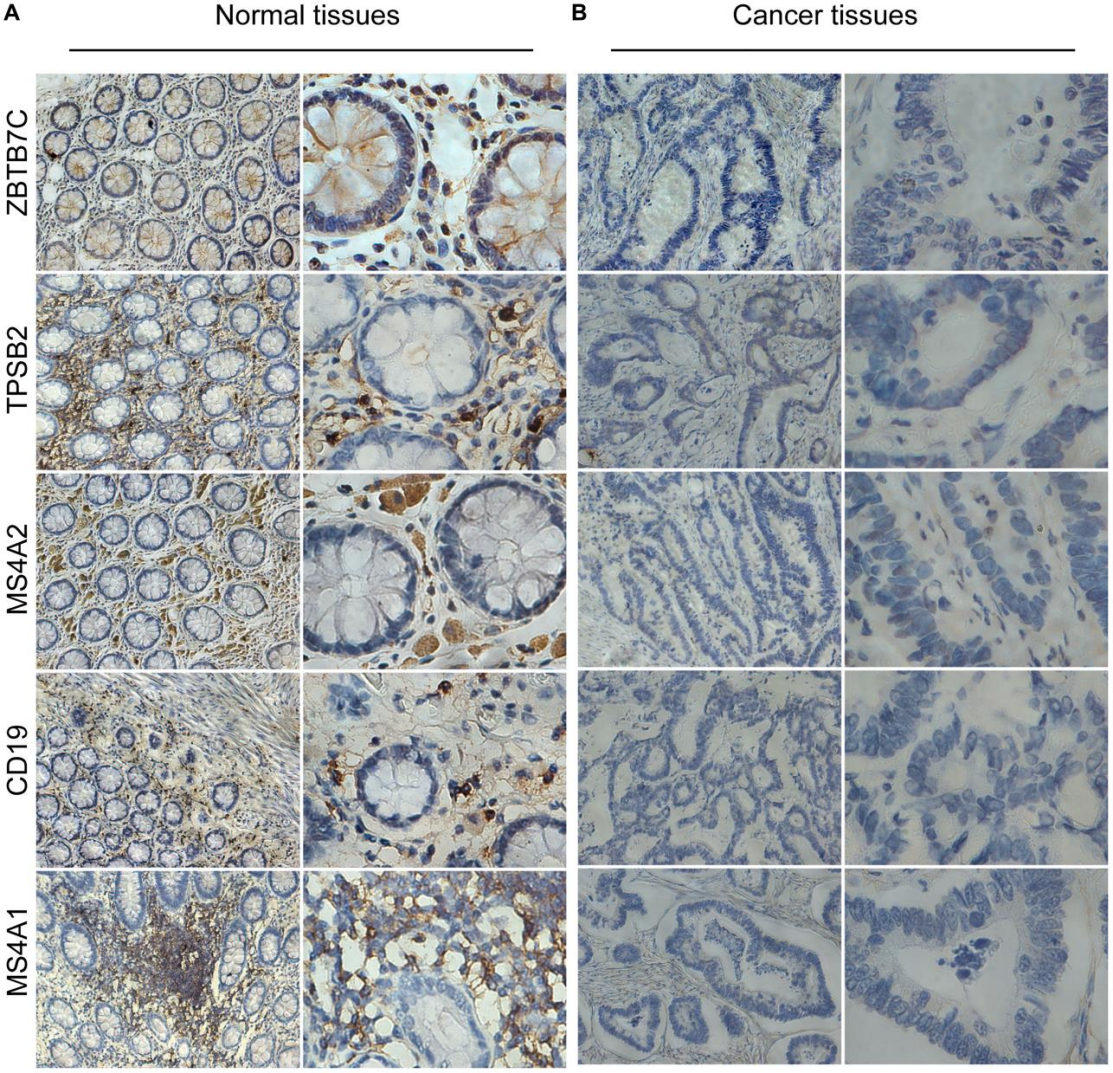
The protein levels of ZBTB7C and gene markers in colorectal cancer (**A**) and adjacent (**B**) tissues.

Next, we used traditional immune cell databases to supplement to the ssGSEA method to further verify the correlations of mast cells and B cells with ZBTB7C. Both Gene Expression Omnibus (GEO) (Figure 5G) and TCGA (Figure 5H) data showed that ZBTB7C had a strong correlation with mast cell markers (TPSB2, CPA3 and MS4A2) and B cell markers (CD19 and MS4A1). RT-qPCR (*n* = 17) and IHC (*n* = 20) were used to detect the mRNA and protein expression levels in CRC and adjacent tissues. At both the mRNA level (Figure 6C) and the protein level (Figure 7; Supplementary Table 3), the levels of ZBTB7C, TPSB2, MS4A2, CD19, and MS4A1 in CRC tissues were lower than those in normal tissues adjacent to the cancer. This finding was consistent with the TCGA (Figure 6A) and GEO (Figure 6B) data results. Finally, the GEPIA database was also used to confirm our conclusion that TPSB2 (Figure 6D), MS4A2 (Figure 6E), CD19 (Figure 6G) and MS4A1 (Figure 6H) showed low expression in COAD and READ.

### The relationship between mast cells and B cells and the prognosis of CRC patients

Kaplan-Meier survival curves based on TCGA clinical information and mast cell and B cell ssGSEA scores were constructed to evaluate prognostic value. We found that groups with higher mast cells had better long-term prognosis than those with lower mast cells (*P* = 0.048 Figure 6F). Similarly, groups with higher B cells also had better long-term prognosis than those with lower B cells (*P* = 0.040 Figure 6I). In summary, mast cells and B cells may be potential protective factors for CRC, and their absence implies poor prognosis.

## DISCUSSION

With the rapid aging of the world's population, major medical, social, and economic problems caused by malignant tumors have emerged. Studies have shown that a main characteristic of aging is mutation accumulation [13]. Aging can increase the incidence of cancer by inducing a series of functional and structural changes in the immune system [14]. In addition, age-related changes in the balance of proinflammatory/anti-inflammatory signals may lead to changes in the TME that make it easier to promote tumor development and growth [15, 16]. As a key transcription factor, ZBTB7C has been reported in many different cancers, but current research shows that its expression and function have strong tissue specificity. To resolve the conflicting data regarding ZBTB7C, we evaluated the general and individual characteristics of ZBTB7C in tumors through a comprehensive pancancer analysis, providing new insights for individualized and precise treatment of tumors.

The pancancer analysis showed that ZBTB7C expression was significantly low in many tumors, suggesting a tumor suppressor effect. ZBTB7C was highly expressed in only LUSC. The mechanism for ZBTB7C expression is not yet clear, and further research is needed. ZBTB7C was also shown to be a protective factor in COAD, DLBC, ESCA and MESO, in which its overexpression was related to superior prognosis. In particular, ZBTB7C expression could be used as an independent prognostic factor in COAD and MESO and could have a major role in prognostic classification.

TMB is an emerging independent biomarker [17, 18] that detects the total number of mutations in the coding region of tumor genes and can be used to stratify the response of patients to immune checkpoint inhibitors (ICPIs). TMB is positively correlated with the efficacy of immunotherapy. Similarly, as an indicator of increased ICPI sensitivity, MSI-H influences in a series of molecular and biological features, including high TMB and altered immune cell infiltration [19]. Most patients with MSI-H tumors tend to show better therapy outcomes and prognosis than MSI-L or MSS patients [2, 20]. In our study, five types of tumors (COAD, SARC, SKCM, PRAD, and BRCA) were related to TMB and MSI and had the same trend. Considering the correlation coefficients^®^ and *P* values comprehensively, ZBTB7C had the strongest correlation with TMB and MSI in COAD. The strong positive correlation in COAD shows that ZBTB7C can be used as a potential biomarker for the efficacy of immunotherapy, supplementing and validating the conclusion of the abovementioned multivariate Cox regression analysis.

To further clarify the relationship between ZBTB7C and immune cell infiltration, the ssGSEA method was used to determine the immune profile in CRC. According to our results, ZBTB7C was positively correlated with the infiltration of most immune cells, especially mast cells and B cells. Mast cells and B cells are unlike other immune cells that have been extensively studied, as there are many knowledge gaps and controversies in the study of mast cells and B cells in tumors. First, as far as mast cells are concerned, some scholars believe that they are involved in the inflammatory response induced by stress and promote tumor angiogenesis and distant metastasis [21, 22]. Other scholars believe that histamine secreted by mast cells can stimulate vascular endothelial cells to produce prostacyclin, which can mediate tumor cell necrosis and inhibit tumor cell metastasis together with TNF-a, IL-1, and IL-6 secreted by mast cells. [23]. Oldford et al. [24] observed that IL-6 secreted by mast cells could activate Toll-like receptor-2 (TLR-2), which can inhibit tumors *in vivo* and *in vitro*. Maltby et al. [25] also found that tryptase and IL-5 secreted by mast cells can promote the recruitment and activation of eosinophils, thereby inhibiting tumor growth. Although B cells can mediate immune suppression and secretion of tumor-promoting factors [26], they more often play a protective role. Studies have shown that tumor-infiltrating B cells can be used as predictors of outcome in metastatic melanoma [27], cervical cancer [28], lung cancer [29, 30] and ovarian cancer [31], and increased infiltration of B cells is associated with superior prognosis.

Obviously, tumor-infiltrating immune cells are affected by tissue specificity and other factors in the TME. Accordingly, we studied the differential expression of mast cells and B cells and their relationship with survival in CRC. The results showed that compared with adjacent tissues, CRC tissues showed low expression of mast cell and B cell markers. In addition, the high expression of markers of these two immune cells suggested a superior prognosis in CRC. Both mast cells and B cells participate in tumor inflammation. This specific inflammatory response has strong tissue specificity, showing a tumor-promoting effect in most tumors and an inhibitory effect in CRC [32]. This phenomenon explains our results very well.

Metabolism and immunity are two major components that cannot be ignored in the tumor microenvironment. Their interaction and effects together induce dynamic changes in the microenvironment. This complex network of intertwined relationships between metabolism and immunity has become a popular topic for in-depth research on the TME. According to previous research by our research group, ZBTB7C is a metabolism-related transcription factor, and Myc showed the strongest negative correlation with it [10]. More importantly, ZBTB7C can also regulate tumor glutamine metabolism by affecting the transcription of glutaminase (GLS1) [11]. Myc is also related to glutamine metabolism [33]. Interestingly, tumor cells and immune cells compete for nutrition in a crowded microenvironment and coexist competitively [34, 35]. Considering these findings and our data, ZBTB7C may inhibit Myc and then inhibit tumor cell glutamine metabolism, resulting in a relative excess of glutamine in the microenvironment, promoting the proliferation of immune cells (especially mast cells and B cells), and ultimately inhibiting the proliferation of CRC cells to achieve a tumor suppressor effect.

However, even though we integrated information from multiple databases and validated our conclusions with clinical samples, this study still has limitations. First, we only studied the immune profile related to ZBTB7C in CRC, as we believed it was the most valuable. Given the tissue specificity of the immune microenvironment, we cannot directly apply our conclusions to all cancers. Second, we need more basic experiments to determine the effects of ZBTB7C on metabolism and immunity. Finally, tissue specificity is not the only thing that needs to be considered; tumor progression may also change the role of the immune microenvironment. Therefore, more evidence is required to supplement our conclusions. Regardless, this article provides directions and ideas for pancancer research on ZBTB7C, and we will improve our study limitations in follow-up research.

## CONCLUSIONS

In summary, we performed a systematic analysis of the expression of ZBTB7C across cancers. This study found for the first time that ZBTB7C is widely underexpressed and is related to prognosis across cancers; in addition, ZBTB7C is related to TMB, MSI and immune infiltration, especially in COAD. ZBTB7C can be used as a biomarker of prognosis across cancers, and it plays an important role in the recruitment and regulation of infiltrating immune cells in CRC. These findings may provide a new tumor immunotherapy target as a bridge between the metabolic microenvironment and the immune microenvironment.

## MATERIALS AND METHODS

### TCGA and GEO databases

The public gene expression profile GSE39582 was downloaded from GEO (http://www.ncbi.nlm.nih.gov/geo) as a verification set. GSE39582 contains data from 566 CRC and 19 noncancerous tissues derived from analyses with an Affymetrix HG-U133 Plus 2.0 chip. The transcriptome, mutation and clinical data of 33 TCGA tumors (10,327 tumor tissues and 730 noncancerous tissues) were obtained from UCSC Xena (https://xena.ucsc.edu/) for pancancer analysis. All data were preprocessed with R4.0.0 software.

### Pancancer analysis

The Ggpubr R package was used to identify and confirm the expression pattern of ZBTB7C across cancers. The survival R package was used to generate the Kaplan-Meier survival curves based on ZBTB7C expression across cancers. The tumor groups associated with prolonged overall survival were selected for univariate and multivariate Cox regression analyses. The Fmsb R package was used to build radar charts for TMB and MSI. The online databases Oncomine (https://www.oncomine.org/) [36], Tumor IMmune Estimation Resource (TIMER) (http://timer.cistrome.org/) [37] and Gene Expression Profiling Interactive Analysis (GEPIA) (http://gepia.cancer-pku.cn/) were used to verify our various conclusions.

### ssGSEA

The RSEM-standardized RNA-seq and microarray datasets of the TCGA COAD (*n* = 521) cohort and READ (*n* = 177) cohort obtained by the abovementioned method were extracted. A total of 698 patients with CRC participated in this study. We obtained a set of marker genes for immune cell types from Bindea et al. [38]. To perform ssGSEA, we used the GSEA program to derive the absolute enrichment score of the gene signature verified by previous experiments. We quantified the infiltration levels of immune cell types by ssGSEA in the gsva R package. ssGSEA applies gene signatures expressed by immune cell populations to individual cancer samples [12]. Our research involved 24 immune phenotypes, including innate immune cells (dendritic cells (DCs), iDCs, activated DCs (aDCs), eosinophils, mast cells, macrophages, natural killer (NK) cells, NK CD56dim cells, NK CD56bright cells, and neutrophils) and acquired immune cells (B cells, Th1 cells, Th2 cells, gamma-delta T (Tgd) cells, CD8+ T cells, T central memory (Tcm) cells, T effector memory (Tem) cells, and T follicular helper (Tfh) cells).

### Patients

This study randomly collected 17 pairs of fresh CRC specimens and paired adjacent benign tissue specimens from the First Affiliated Hospital of Chongqing Medical University (Chongqing, China) from January to June 2020 for RT-qPCR. Twenty pairs of CRC tissue and normal tissue paraffin sections (collected from June 2018 to March 2019) provided by the Pathology Department of the First Affiliated Hospital of Chongqing Medical University were used for protein level determination. All patients signed an informed consent form, and the study was supported by the Ethics Committee of the First Affiliated Hospital of Chongqing Medical University.

### RT-qPCR

Using TRIzol reagent (Takara Biotechnology Co., Ltd., Dalian, China), according to the manufacturer’s instructions, total RNA was extracted from 17 pairs of fresh CRC and adjacent tissues. Total RNA was reverse transcribed into cDNA using a PrimeScript™ RT kit (Takara Biotechnology Co., Ltd.). Then, PCR was performed according to the instructions provided by the qPCR reagents. The primers were designed by Takara Biotechnology Co., Ltd. The sequences were as follows: ZBTB7C: forward, 5′-CCACGAACTACCTTCAACTCC-3′, and reverse, 5′-GTGATCTCCTTCTGCATCCTGT-3′; TPSB2: forward, 5′-CGGCGGCGCACTGT-3′, and reverse, 5′-GCAGCTGCACCCTGATGTCTCT-3′; MS4A2: forward, 5′-AACACTGCCAGCAGCATAGC-3′, and reverse, 5′-AAGCCATAAAGCATTGGTCTC-3′; CD19: forward, 5′-CTTTGGCTTATCTGATCTTCTGC-3', and reverse, 5′ -GGGGTCAGTCATTCGCTTTC-3′; MS4A1: forward, 5′-CAGGAACTTGTAATAGCTGGCAT-3′, and reverse, 5′-GGTTGGGAAGATGTTTCAGTTAG-3′; and β-actin: forward, 5′-AGAAAATCTGGCACCACACCT-3′, and reverse, 5′-GATAGCACAGCCTGGATAGCA-3′. The expression was normalized to that of β-actin, and the relative expression was calculated using the 2-ΔΔCt method [39].

### IHC

According to a previously reported method [10], 20 pairs of CRC and paracarcinoma paraffin sections were immunohistochemically stained. The slices were incubated with primary antibodies against ZBTB7C (BS-13583R; 1:100), TPSB2 (AB232787; 1:200), MS4A2 (AB203747; 1:100), CD19 (SC-19650; 1:200) and MS4A1 (SC-393894; 1:200). The images were captured using a Leica microscope imaging system (100× and 400× magnification; Leica Microsystems GM-bh, Wetzlar, Germany). The data were evaluated by two independent pathologists who were blinded to the sample information. A semiquantitative immune response scoring system was used to divide patients into high expression groups and low expression groups according to their immune response scores [40, 41].

### Statistical analysis

R 4.0.0 and SPSS 25.0 software were used for statistical analysis. GraphPad Prism 8.0.2 and R 4.0.0 were used to plot the statistical results. All experiments were performed in triplicate. A chi-square test was performed on the IHC data to analyze the relationship between ZBTB7C expression and the clinicopathological parameters of patients with CRC. The Spearman method was used to calculate correlations. Student's *t* test was used to distinguish differences between groups. A two-tailed test resulting in *P* < 0.05 was considered to indicate a statistically significant difference.

## Supplementary Materials

Supplementary Tables
