# Supplementary material for: The prognostic and immunological effects of ZBTB7C across cancers: friend or foe?
Source: Aging (Albany NY). 2021 May 4;13(9):12849–64. doi: 10.18632/aging.202955 (PMC8148469; doi:10.18632/aging.202955)
Supplement: Supplementary Tables [file aging-13-202955-s001.pdf]

SUPPLEMENTARY TABLES

Supplementary Table 1. Correlation between ZBTB7C and TMB in pancancer.

CancerType	Cor	*P*
ACC	0.074986763	0.511310077
BLCA	–0.023807658	0.631594569
BRCA	–0.137956886	***
CESC	–0.070600864	0.233957663
CHOL	–0.167074652	0.330087276
COAD	0.2088901	***
DLBC	–0.210052157	0.211311022
ESCA	–0.156532988	*
GBM	–0.021331606	0.796917012
HNSC	0.090829459	*
KICH	0.12121683	0.336113993
KIRC	–0.047641365	0.386882701
KIRP	–0.059855833	0.320032389
LAML	0.113883384	0.374155484
LGG	–0.070672552	0.114131961
LIHC	–0.073222316	0.166240488
LUAD	–0.39699556	***
LUSC	–0.098686912	*
MESO	0.005856676	0.959145448
OV	–0.074681008	0.219554492
PAAD	0.246420436	**
PCPG	–0.018569788	0.806204266
PRAD	–0.361925354	***
READ	0.002082146	0.98109627
SARC	–0.220854452	***
SKCM	–0.263837157	***
STAD	–0.011832685	0.821025702
TGCT	–0.108910668	0.192237047
THCA	–0.003019664	0.947279711
THYM	0.375722423	***
UCEC	–0.052537446	0.229462386
UCS	–0.045716441	0.737949911
UVM	–0.06837225	0.54676078

Cor, R value of Spearman's correlation. $^{*}P < 0.05$; $^{**}P < 0.01$; $^{***}P < 0.001$.

Supplementary Table 2. Correlation between ZBTB7C and MSI in pancancer.

CancerType	Cor	*P*
ACC	0.078433282	0.492033508
BLCA	–0.104057123	*
BRCA	–0.093148164	**
CESC	–0.078290524	0.174785725
CHOL	–0.293436293	0.082659076

COAD	0.198824035	***
DLBC	–0.053630072	0.717332196
ESCA	–0.104985977	0.186427612
GBM	0.161360935	*
HNSC	–0.013970642	0.756278789
KICH	0.130956935	0.298428825
KIRC	–0.024393693	0.656409899
KIRP	–0.074899789	0.207428345
LAML	–0.188648273	0.12339913
LGG	–0.002708062	0.951449703
LIHC	–0.077939833	0.135079037
LUAD	–0.036561006	0.409527959
LUSC	0.005661489	0.900216894
MESO	–0.134076389	0.229787922
OV	–0.012422068	0.838404792
PAAD	–0.098395235	0.195160086
PCPG	–0.069253623	0.358329713
PRAD	–0.13430933	**
READ	–0.020843265	0.798816238
SARC	–0.145062633	*
SKCM	–0.132410305	**
STAD	0.037459798	0.470130607
TGCT	0.00593466	0.9425406
THCA	–0.046066899	0.308339037
THYM	0.084963167	0.360314041
UCEC	0.000573876	0.989404411
UCS	–0.169178714	0.212591461
UVM	0.090092308	0.426761343

Cor, R value of Spearman's correlation. $^{*}P < 0.05$; $^{**}P < 0.01$; $^{***}P < 0.001$.

Supplementary Table 3. Immunohistochemical analysis of ZBTB7C and gene markers in colorectal cancer and adjacent tissues.

	tissue		***P* value (χ^2)**
	**Normal (*n* = 20)**	**Cancer (*n* = 20)**	
Immunohistochemical grade			
Low expression ZBTB7C	1 (5%)	13 (65%)	<0.001
High expression ZBTB7C	19 (95%)	7 (35%)	
Low expression TPSB2	4 (20%)	12 (60%)	0.010
High expression TPSB2	16 (80%)	8 (40%)	
Low expression MS4A2	2 (10%)	10 (50%)	0.006
High expression MS4A2	18 (90%)	10 (50%)	
Low expression CD19	8 (40%)	16 (80%)	0.010
High expression CD19	12 (60%)	4 (20%)	
Low expression MS4A1	6 (30%)	13 (65%)	0.027
High expression MS4A1	14 (70%)	7 (35%)	
